# Spherical Hybrid Nanoparticles for Homann Stagnation-Point Flow in Porous Media via Homotopy Analysis Method

**DOI:** 10.3390/nano13061000

**Published:** 2023-03-09

**Authors:** Xiangcheng You, Jifeng Cui

**Affiliations:** 1 State Key Laboratory of Petroleum Resources and Prospecting, College of Petroleum Engineering, University of Petroleum (Beijing), Beijing 102249, China; xcyou@cup.edu.cn; 2 College of Sciences, Inner Mongolia University of Technology, Hohhot 010051, China

**Keywords:** spherical hybrid nanoparticles, Homann stagnation-point flow, homotopy analysis method (HAM), porous media

## Abstract

Non-axisymmetric stagnant-point flows for flat plates in porous media containing spherical Cu-Al_2_O_3_-H_2_O nanoparticles are studied using the homotopy analysis method (HAM). The governing equations are transformed into three coupled non-linear ordinary differential equations through similarity transformations. A large degree of freedom is provided by HAM when selecting auxiliary linear operators. By transforming nonlinear coupled ordinary differential equations with variable coefficients into linear ordinary differential equations with constant coefficients, nonlinear coupled ordinary differential equations can be solved. Over the entire domain, these equations can be solved approximately analytically. The analysis involves a discussion of the impact of many physical parameters generated in the proposed model. The results have shown that skin friction coefficients of *Cf_x_* and *Cf_y_* increase with volume fraction of hybrid nanofluid and the coefficient of permeability increasing. For the axisymmetric case of *γ* = 0, when volume fraction, *φ*, *φ*_1_, *φ*_2_ = 0, 5%, 10%, 20%, *Cf_x_* = *Cf_y_* = 1.33634, 1.51918, 1.73905, 2.33449, it can be found that the wall shear stress values increase by 13.68%, 30.14%, and 74.69%, respectively. In response to an increase in hybrid nanofluid volume fractions, local Nusselt numbers *Nu_x_* increase. *Nu_x_* decrease and change clearly with the coefficient of permeability increasing in the range of *γ* < 0; the values of Nu_x_ are less affected in the range of *γ* > 0.

## 1. Introduction

In the analysis of fluid dynamics, properties of heat transfer have always been the focus of research, especially in the field of engineering, where enhancing heat transfer is very important. Hybrid nanofluids have been proposed and verified using thermal productivity calculations based on numerical and experimental results. Mixed convective Al_2_O_3_-water nanofluids were studied experimentally on inclined copper tubes by Momin et al. [1]. Studies and discussions of how nanoparticle concentration and power supply affect laminar flow thermal fields have been conducted. In a two-stage procedure, Sahoo et al. [2] prepared (Al_2_O_3_-SiC-TiO_2_) ternary hybrid nanofluids based on water. As a result of this analysis, Al_2_O_3_-SiC-TiO_2_ nanoparticles were equally distributed across each volume fraction sample of ternary hybrid nanofluids. Using numerical simulations and experiments, Zufar et al. [3] studied heat pipes and found that those including hybrid nanofluids perform well thermally. Al_2_O_3_-CuO and SiO_2_-CuO hybrid nanofluids were characterized with respect to their thermodynamic conductivity and viscosity. Hybrid nanofluid flows with coupling stresses on the tensile surface were examined by Saeed et al. [4] using the homotopy analysis method. Nasir et al. [5] considered the effects of nonlinear thermal radiation on nanofluids based on water, hybrid, and ternary hybrids on stretched sheets using the homotopy analysis method. Hybrid nanofluids were used experimentally as heat transfer mediums in collectors and storage systems for solar energy applications by Yasmin et al. [6].

It is possible to improve the convective heat transfer characteristics of processes by using hybrid nanofluids in porous media, such as geothermal, oil flow filtration, all types of heat exchangers, and so on. A variety of hybrid nanofluids for a variety of applications showed increased efficiency and energy savings. Various aspects of hybrid nanofluids, including their synthesis, thermophysics, and heat transfer, have recently been reviewed by Sarkar et al. [7] and Babu et al. [8]. Although hybrid nanofluids might transfer heat better with proper hybridization, problems with preparation and stability still need to be overcome. Kasaeian et al. [9] conducted a comprehensive review of nanofluids and porous media for improving a thermal system’s heat transfer with varying structures and flow states. Das et al. [10] thoroughly reviewed experiments and numerical research on different nanofluids, as well as the most recent advancements in the study of thermal conductivity. Additionally, nanoparticles with a variety of types and sizes, solid volume fractions, different types of basic fluids, temperatures, and various mechanisms are discussed, all of which influence thermal conductivity [11,12]. A number of models and media were proposed by Tiwar et al. [13] for study of the thermal parameters of solar collectors in presence of nanofluids. Fluids used for heat transfer that consist of nanofluids and hybrid nanofluids can enhance the efficiency of solar collector absorption tubes. Sangapatanam et al. [14] presented a review of hybrid nanofluids’ preparation, stability, and characteristics of thermophysics. Moreover, how to control the pH and ultrasonic intensity of hybrid nanofluids has been discussed. Research on the effects of nanofluid type, nanoparticle concentration, and nanofluid depth on solar cell performance was reviewed by Modi et al. [15]. How nanofluids can be applied to solar cells and various enhancement techniques have also been discussed [16]. As part of their experimental investigation, Jana et al. [17] explored the impact of hybrid nanoparticles on fluid heat transfer. Mechanisms for improving thermal conductivity and the effects of nanoparticles on stability were also discussed. According to Suresh et al. [18], hybrid nanofluids exhibited an increase in thermal conductivity with increasing concentrations, and they found that the increase in viscosity was much higher than of the increase in thermal conductivity. Takabi et al. [19] numerically studied the effects of hybrid nanofluids as working fluids on thermal properties of a shell and its temperature field. Devi et al. [20] studied flows and heat transfer phenomena using hybrid nanofluids under magnetic fields on permeable tensile sheets. Hybrid nanofluids that improve the performance of suspended nanoparticles were discussed by Nabil et al. [21]. Bibi et al. [22] investigated flows and heat transfer of Cu, Al_2_O_3_, and TiO_2_-H_2_O nanofluids in vertical planes of anisotropic permeable saturated porous media at high Rayleigh numbers. Hayat et al. [23] considered nanoparticle volume fraction, magnetohydrodynamics, and heat generation–absorption effects on hybrid nanofluid flows through a stretched surface, and also compared the heat transfer properties of conventional and novel hybrid nanofluids. Saeed et al. [24] studied the thermal characteristics of Darcy–Forchheimer hydromagnetic hybrid nanofluids flowing in permeable stretch cylinders, including Brownian motion and the thermophoresis effect. In a porous medium, Wahid et al. [25] numerically simulated flows of Marangoni hybrid nanofluids, demonstrating the importance of porous media and hybrid nanofluids. Using magnetic fields and heat sources/sinks, Othman et al. [26] investigated the flows and heat transfer behaviors of carbon nanotubes on permeable exponentially shrinking surfaces. Khan et al. [27] considered the radiation-mixed convection flows of hybrid nanofluids through vertical cylinders with porous heat sources and sinks. Rostami et al. [28] studied the flows of mixture-based dusty hybrid nanofluids on stretched plates in porous media under action of magnetic fields, and concluded that heat transfer efficiency could be increased by improving thermal and electrical conductivity. An investigation into the free convection of hybrid nanosuspensions in geometrically triangular porous vessels with internal heat generation, inclined Lorentz force, and radiation was conducted by Ahmed et al. [29]. A significant part of the heat transfer efficiency and fluid flow is determined by the heated and wavy walls within the chamber. Porous infinite plates were used by Chu et al. [30] to convey the thermal effects of hybrid nanofluids on different nanoparticles. Copper (Cu), titanium oxide (TiO_2_), and aluminum oxide (Al_2_O_3_) with water-based liquids were used in the characterization of hybrid nanoparticles.

There are many references on stagnation point flow, some of which are given below. Ariel [31] studied the two-dimensional stagnation-point flow problem of non-Newtonian liquids. Mahapatra et al. [32,33] considered the steady stagnation flows of viscoelastic liquids on the stretched boundary, and obtained similar solutions of the stagnation flows of magnetic fluids. Weidman [34,35] investigated a modification of Homann’s axisymmetric stagnation-point flows, and compared it to a rigid plate so that it was non-axisymmetric. By applying the homotopy analysis method, Nawaz et al. [36] studied radially stretched thin plates causing laminar boundary layer flows of nanofluids, considering thermophoresis and Brownian motion. Azam et al. [37] numerically studied the unsteady magnetic fluid stagnation-point flows of Carreau nanofluids on the expansion/contraction cylinder under the action of nonlinear thermal radiation, taking into account the condition that the mass flux of nanoparticles at the boundary of the Buongiorno model is zero. Ahmed et al. [38] considered rotational stagnation flows of Maxwell nanofluids in porous rotating disks. Modified Buongiorno nanofluid models were used to investigate the effects of variable thermal conductivities and heat sources on heat transfer characteristics. On porous stretched/contracted plates, Kho et al. [39] studied two-dimensional Hermann stagnation-point flows and heat transfer of hybrid nanofluids. Numerical analysis of hybrid nanofluid flows in porous media in a non-axisymmetric stagnation region was conducted by Waini et al. [40].

A wide range of problems involving strong nonlinearity can be solved with the homotopy analysis method (HAM) [41]. The HAM method cannot rely on small or large physical parameters to produce results, in contrast with perturbation methods. Higher-order approximate series solutions can also be selected via HAM using its expression form [42,43]. It is also possible to use HAM to simply control the convergence of series solutions. With regard to analytic and semi-analytic methods for nonlinear partial differential equations, HAM demonstrates the unusual convergence characteristics of series solutions [44,45]. The homotopy analysis method was used by Xu et al. [46] to investigate the growth of velocity boundary layers caused by pulse initiation wedges. Analytical approximations of momentum boundary layers were obtained that matched Keller box numerical results. With arbitrary stretching velocity on a flat plate, You et al. [47] investigated non-similar boundary layer flows of second-order fluids. Based on the results of HAM, local skin friction coefficients, local Nusselt numbers, and boundary layer thicknesses were considered in detail [48]. According to Sardanyés et al. [49], homotopy solutions were obtained for a nonlinear model of cancer that described the interaction of tumor cells with healthy and immune cells. Mathematica software package BVPH 2.0 with HAM was used by Farooq et al. [50] to solve the magnetic hydrodynamics Falkner–Skan flow of nanofluids in a semi-infinite domain. In the stagnation region, Mustafa et al. [51] observed Carson fluid flowing to the stretched sheet. A homotopy analysis solution was obtained by analyzing the entire space domain. Heat transfer characteristics with viscous dissipation were also analyzed. In a porous medium facing an exponentially stretched porous plate, Ali et al. [52] presented homotopy analytical solutions for boundary layers and heat transfer flow. Researchers studied magnetohydrodynamic secondary nanofluid flows through biaxially stretched surfaces in the work of Ramzan et al. [53]. In unsaturated porous media, Patel et al. [54] used the homotopy analysis method to give the nonlinear one-dimensional solution to Boussinesq’s equation. A magneticohydrodynamic biovector Walter-B nanofluid flow caused by stretched thin plates was considered by Hayat et al. [55]. A combination of the melting parameter, radiation parameter, magnetic parameter, Brownian motion parameter, Prandtl number, Peclet number, Dufour number, and Soret number was investigated. Using a nonlinear reaction–diffusion system, Al-Qudaha et al. [56] proposed an optimal homotopy analysis algorithm. In this linearization algorithm, homotopy series solutions are constructed by using Taylor series approximation of nonlinear equations. The results showed that linearization improved homotopy series solution accuracy and convergence. Bottona et al. [57] mathematically analyzed the vertical movement of two different spherical non-evaporative droplets along the centerline. Taking into account Blasius viscous flow, magnetohydrodynamic flow, boundary layer flows resulting from free convection, and Von Kármán swirling viscous flow, a comparison of homotopy renormalization (HTR) and homotopy analysis (HAM) was made by Yang et al. [58]. Similarly, it was found that HAM approximation was significantly more accurate than HTR approximation. Magnetohydrodynamic flows and heat transfer analysis of Casson fluids on an exponentially shrinking thin plate were carried out by Liu et al. [59]. To solve the forced Duffing equation, Prof. Liao [60] used the non-perturbation method based on homotopy analysis, namely “Directly Defining Inverse Mapping” (MDDiM). With this algorithm, all small denominators were completely avoided, and multiple limit cycles of the forced Duffing equation with high nonlinearity could be obtained successfully.

Using the homotopy analysis method (HAM), this study approximates analytical solutions for hybrid nanofluids flowing through flat sheets at a Homann stationary-point. With the introduction in Section 1, Section 2 presents the mathematical solution and derivative using HAM. A discussion and graphic illustration of results is included in Section 3. Lastly, a summary of the main findings is included in Section 4.

## 2. Mathematical Model and Method

**Figure 1:**
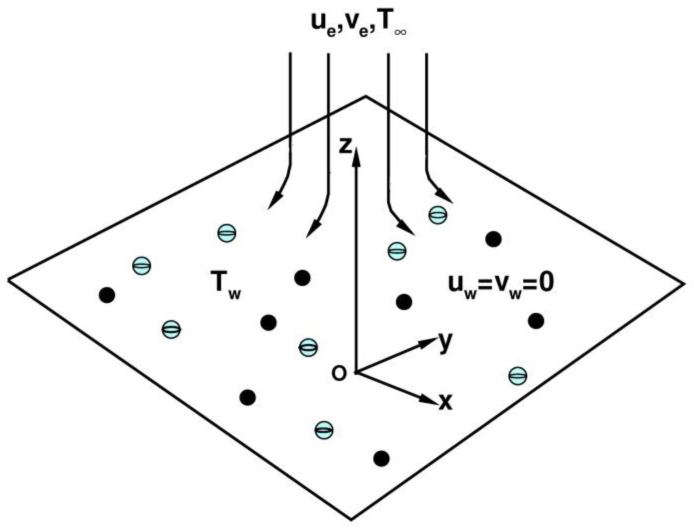
Physical model and coordinate systems.

The Cu-Al_2_O_3_-H_2_O hybrid nanofluid model for Hohmann stagnation-point flows in porous media is shown in Figure 1, where *z* is the normal direction, and *xy* is the plane in Cartesian coordinate systems. The governing equations are (Weidman [34,35]; Waini et al. [40]):

(1) $$\frac{\partial \bar{u}}{\partial \bar{x}}+\frac{\partial \bar{v}}{\partial \bar{y}}+\frac{\partial \bar{w}}{\partial \bar{z}}=0,$$

(2) $$\bar{u}\frac{\partial \bar{u}}{\partial \bar{x}}+\bar{v}\frac{\partial \bar{u}}{\partial \bar{y}}+\bar{w}\frac{\partial \bar{u}}{\partial \bar{z}}={\bar{u}}_{e}\frac{d{\bar{u}}_{e}}{d\bar{x}}+\frac{\mu_{hnf}}{\rho_{hnf}}\frac{\partial^{2}\bar{u}}{\partial {\bar{z}}^{2}}-\frac{\mu_{hnf}}{K\rho_{hnf}}\left(\bar{u}-{\bar{u}}_{e}\right),$$

(3) $$\bar{u}\frac{\partial \bar{v}}{\partial \bar{x}}+\bar{v}\frac{\partial \bar{v}}{\partial \bar{y}}+\bar{w}\frac{\partial \bar{v}}{\partial \bar{z}}={\bar{v}}_{e}\frac{d{\bar{v}}_{e}}{d\bar{x}}+\frac{\mu_{hnf}}{\rho_{hnf}}\frac{\partial^{2}\bar{v}}{\partial {\bar{z}}^{2}}-\frac{\mu_{hnf}}{\bar{K}\rho_{hnf}}\left(\bar{v}-{\bar{v}}_{e}\right),$$

(4) $$\bar{u}\frac{\partial \bar{T}}{\partial \bar{x}}+\bar{v}\frac{\partial \bar{T}}{\partial \bar{y}}+\bar{w}\frac{\partial \bar{T}}{\partial \bar{z}}=\frac{{\bar{k}}_{hnf}}{{\left(\rho c_{p}\right)}_{hnf}}\frac{\partial^{2}\bar{T}}{\partial {\bar{z}}^{2}},$$

Assuming

(5) $$\begin{array}{l} \bar{u}=0,\bar{v}=0,\bar{w}=0,\bar{T}=\begin{array}{cc} {\bar{T}}_{w}\left(x\right) & \begin{array}{cc} at & \bar{z}= \end{array} \end{array}0\\ \bar{u}\to {\bar{u}}_{e}\left(x\right),\bar{v}\to {\bar{v}}_{e}\left(x\right),\bar{w}\to {\bar{w}}_{e}\left(x\right),\begin{array}{cc} \bar{T}\to {\bar{T}}_{\infty } & \begin{array}{cc} at & \bar{z}\to \end{array} \end{array}\infty , \end{array}$$

In the formula, $\bar{u},\bar{v},\bar{w}$ are the velocity components, the external flow velocities are ${\bar{u}}_{e}\left(\bar{x},\bar{y}\right)=\bar{x}\left(\bar{a}+\bar{b}\right)$, ${\bar{v}}_{e}\left(\bar{x},\bar{y}\right)=\bar{y}\left(\bar{a}-\bar{b}\right)$, ${\bar{w}}_{e}\left(\bar{z}\right)=-2\bar{a}\bar{z}$, where $\bar{a},\bar{b}$ are shear-strain rates. The surface temperature is ${\bar{T}}_{w}={\bar{T}}_{\infty }+{\bar{T}}_{0}\bar{x}$ (${\bar{T}}_{\infty }$ ambient temperature, ${\bar{T}}_{0}$ characteristic temperature), $\bar{K}$ represents the permeability of porous media. $\bar{k}$ means the conductivity in temperature, ρ represents fluid density, *μ* is dynamic viscosity, and $c_{p}$ means specific heat capacity coefficient. Table 1 shows thermophysical properties of pure water, Cu, and Al_2_O_3_ nanoparticles. Table 2 provides the applied model for the thermophysical properties of spherical hybrid nanoparticles. The symbols $\phi_{1}$ and $\phi_{2}$ represent Cu and Al_2_O_3_ nanoparticles, respectively, and $\phi =\phi_{1}+\phi_{2}$, where solid compositions are represented by subscripts n1 and n2.

Using the similarity transformation (Weidman [34,35]; Waini et al. [40]):

(6) $$\begin{array}{l} \bar{u}=\bar{x}f^{\prime }\left(\eta \right)\left(\bar{a}+\bar{b}\right),\bar{v}=\bar{y}g^{\prime }\left(\eta \right)\left(\bar{a}-\bar{b}\right),\bar{w}=-\sqrt{\bar{a}\nu_{f}} [f\left(\eta \right)\left(\bar{a}+\bar{b}\right)+g\left(\eta \right)\left(\bar{a}-\bar{b}\right)]\\ \theta =\frac{\bar{T}-{\bar{T}}_{\infty }}{{\bar{T}}_{w}-{\bar{T}}_{\infty }},\eta =\bar{z}\sqrt{\frac{\bar{a}}{\nu_{f}}} \end{array},$$

In the formula, ‘ means the derivative of *η*.

Substitute Equation (6) into Equations (1)–(4), and one has:

(7) $$A_{1}f^{\prime \prime \prime }+\left(1+\gamma \right)\left(ff^{\prime \prime }+1-{f^{\prime }}^{2}\right)+\left(1-\gamma \right)gf^{\prime \prime }-A_{1}\sigma \left(f^{\prime }-1\right)=0,$$

(8) $$A_{1}g^{\prime \prime \prime }+\left(1-\gamma \right)\left(gg^{\prime \prime }+1-{g^{\prime }}^{2}\right)+\left(1+\gamma \right)fg^{\prime \prime }-A_{1}\sigma \left(g^{\prime }-1\right)=0,$$

(9) $$\frac{A_{2}}{\mathrm{Pr}}\theta^{\prime \prime }+\left(1+\gamma \right)\left(f\theta^{\prime }-f^{\prime }\theta \right)+\left(1-\gamma \right)g\theta^{\prime }=0,$$

depending on

(10) $$\begin{array}{l} f\left(0\right)=0,f^{\prime }\left(0\right)=0,g\left(0\right)=0,g^{\prime }\left(0\right)=0,\theta \left(0\right)=1\\ f^{\prime }\left(\eta \right)\to 1,g^{\prime }\left(\eta \right)\to 1,\theta \left(\eta \right)\to 0\begin{array}{c} \begin{array}{cc} at & \eta \to \end{array} \end{array}\infty , \end{array}$$

In the formula, the reflective symmetries are obtained as $f\left(\eta ,\gamma \right)=g\left(\eta ,-\gamma \right)$ or $f\left(\eta ,-\gamma \right)=g\left(\eta ,\gamma \right)$, where *γ* is the ratio of strain shear rate. The coefficients of $A_{1},A_{2}$, σ, and Pr are given by

(11) $$A_{1}=\frac{\mu_{hnf}/\mu_{f}}{\rho_{hnf}/\rho_{f}},A_{2}=\frac{k_{hnf}/k_{f}}{{\left(\rho c_{p}\right)}_{hnf}/{\left(\rho c_{p}\right)}_{f}},\gamma =\frac{b}{a},\sigma =\frac{\nu_{f}}{aK},\mathrm{Pr}=\frac{\mu_{f}{\left(c_{p}\right)}_{f}}{k_{f}}.$$

The skin friction coefficients of $Cf_{x},Cf_{y}$ and the local Nusselt numbers $Nu_{x}$ are

(12) $${\bar{C}}_{fx}=\frac{\mu_{hnf}}{\rho_{f}u_{e}^{2}}{\left(\frac{\partial \bar{u}}{\partial \bar{z}}\right)}_{\bar{z}=0},{\bar{C}}_{fy}=\frac{\mu_{hnf}}{\rho_{f}v_{e}^{2}}{\left(\frac{\partial \bar{v}}{\partial \bar{z}}\right)}_{\bar{z}=0},{\bar{Nu}}_{x}=-\frac{\bar{x}k_{hnf}}{k_{f}\left({\bar{T}}_{w}-{\bar{T}}_{\infty }\right)}{\left(\frac{\partial \bar{T}}{\partial \bar{z}}\right)}_{\bar{z}=0},$$

(13) Cfx=Rex1+γC¯fx=μhnfμff″0,Cfy=Rey1−γC¯fy=μhnfμfg″0,Nux=1+γRexNu¯x=−khnfkfθ″0,

In the formula, the local Reynolds numbers are Rex=uex/νf, Rey=vey/νf.

With HAM, it is possible to simplify nonlinear problems into linear ones in an infinite number of ways. It is generally accepted that boundary layer flows degenerate exponentially after infinity [61,62]. Based on Equations (13)–(14), $f\left(\eta \right),\theta \left(\eta \right)$ could be expressed by

(14) $$f(\eta )=\sum_{t=0}^{+\infty }\sum_{s=0}^{+\infty }a_{t,s}\eta^{t}e^{-\beta s\eta },g(\eta )=\sum_{t=0}^{+\infty }\sum_{s=0}^{+\infty }b_{t,s}\eta^{t}e^{-\beta s\eta },\theta (\eta )=\sum_{t=0}^{+\infty }\sum_{s=0}^{+\infty }c_{t,s}\eta^{t}e^{-\beta s\eta },$$

In the formula, $a_{t,s},b_{t,s},c_{t,s}$ are constant coefficients determined by HAM.

Equation (14) provides a convenient way to select initial guesses using Equation (10)

(15) $$f_{0}(\eta )=\eta -\frac{1-e^{-\beta \eta }}{\beta },g_{0}(\eta )=\eta -\frac{1-e^{-\beta \eta }}{\beta },\theta_{0}(\eta )=e^{-\beta \eta },$$

with auxiliary linear operators

(16) $$L_{f}=\frac{\partial^{3}F}{\partial \eta^{3}}-\beta^{2}\frac{\partial F}{\partial \eta },L_{g}=\frac{\partial^{3}G}{\partial \eta^{3G}}-\beta^{2}\frac{\partial G}{\partial \eta },L_{\theta }=\frac{\partial^{2}\Theta }{\partial \eta^{2}}-\beta^{2}\Theta ,$$

having the following properties:

(17) $$\begin{array}{l} L_{f} [C_{0}+C_{1}e^{-\beta \eta }+C_{2}e^{\beta \eta }]=0\\ L_{g} [C_{3}+C_{4}e^{-\beta \eta }+C_{5}e^{\beta \eta }]=0,\\ L_{\theta } [C_{6}\exp e^{-\beta \eta }+C_{7}e^{\beta \eta }]=0 \end{array}$$

In the formula, *C*_0_–*C*_7_ are integral coefficients.

HAM deformation equations are constructed:

(18) $$\begin{array}{l} \left(1-q\right)L_{f} [F\left(\eta ;q\right)-f_{0}\left(\eta \right)]=qh_{f}N_{f} [F\left(\eta ;q\right)]\\ \left(1-q\right)L_{g} [G\left(\eta ;q\right)-g_{0}\left(\eta \right)]=qh_{g}N_{g} [G\left(\eta ;q\right)],\\ \left(1-q\right)L_{\theta } [\Theta \left(\eta ;q\right)-\theta_{0}\left(\eta \right)]=qh_{\theta }N_{\theta } [\Theta \left(\eta ;q\right)] \end{array}$$

and

(19) $$\begin{array}{l} F\left(0;q\right)=0,F^{\prime }\left(0;q\right)=0,F^{\prime }\left(\infty ;q\right)=1\\ G\left(0;q\right)=0,G^{\prime }\left(0;q\right)=0,G^{\prime }\left(\infty ;q\right)=1,\\ \Theta \left(0;q\right)=1,\Theta^{\prime }\left(\infty ;q\right)=0 \end{array}$$

In the formula embedding parameter q∈ [0,1], the nonlinear operator based on governing Equations (7)–(9) is $N_{f},N_{g},N_{\theta }$. Using HAM, approximate analytically solutions can be fully determined:

(20) $$\begin{array}{l} F\left(\eta ;0\right)=f_{0}\left(\eta \right),\;\begin{array}{c} F\left(\eta ;1\right)=f\left(\eta \right) \end{array}\\ G\left(\eta ;0\right)=g_{0}\left(\eta \right),\;\begin{array}{c} G\left(\eta ;1\right)=g\left(\eta \right). \end{array}\\ \Theta \left(\eta ;0\right)=\theta_{0}\left(\eta \right),\;\begin{array}{c} \Theta \left(\eta ;1\right)=\theta \left(\eta \right) \end{array} \end{array}$$

*Q* contributes to mapping; as *q* changes from 0 to 1, mapping ensures that $F\left(\eta ;q\right),G\left(\eta ;q\right),\Theta \left(\eta ;q\right)$ deform continuously to exact solutions $f\left(\eta \right),g\left(\eta \right),\theta \left(\eta \right)$ from initial guesses $f_{0}\left(\eta \right),g_{0}\left(\eta \right),\theta_{0}\left(\eta \right)$. By Taylor’s theorem, $F\left(\eta ;q\right),G\left(\eta ;q\right),\Theta \left(\eta ;q\right)$ for power series expansion are:

(21) $$\begin{array}{l} F\left(\eta ;q\right)=F\left(\eta ;0\right)+\sum_{s=1}^{+\infty }f_{s}\left(\eta \right)q^{s}\\ G\left(\eta ;q\right)=G\left(\eta ;0\right)+\sum_{s=1}^{+\infty }g_{s}\left(\eta \right)q^{s},\\ \Theta \left(\eta ;q\right)=\Theta \left(\eta ;0\right)+\sum_{s=1}^{+\infty }\theta_{s}\left(\eta \right)q^{s} \end{array}$$

In the formula

(22) $$f_{s}\left(\eta \right)={\left.\frac{1}{s!}\frac{\partial^{s}G\left(\eta ;q\right)}{\partial q^{s}}\right|}_{q=0},g_{s}\left(\eta \right)={\left.\frac{1}{s!}\frac{\partial^{s}G\left(\eta ;q\right)}{\partial q^{s}}\right|}_{q=0},\theta_{s}\left(\eta \right)={\left.\frac{1}{s!}\frac{\partial^{s}G\left(\eta ;q\right)}{\partial q^{s}}\right|}_{q=0}.$$

Series (21) can be substituted into zero order deformation Equation (18) and boundary Condition (19) as follows. In order to obtain *m* order deformation equation, equal power coefficients of *q* are used:

(23) $$\begin{array}{l} L_{f} [g\left(\eta \right)-\chi_{m}f_{m-1}\left(\eta \right)]=h_{f}R_{fm}\left(\eta \right),\;\begin{array}{c} m\geq 1 \end{array}\\ L_{g} [g\left(\eta \right)-\chi_{m}g_{m-1}\left(\eta \right)]=h_{g}R_{gm}\left(\eta \right),\;\begin{array}{c} m\geq 1, \end{array}\\ L_{\theta } [g\left(\eta \right)-\chi_{m}\theta_{m-1}\left(\eta \right)]=h_{\theta }R_{\theta m}\left(\eta \right),\;\begin{array}{c} m\geq 1 \end{array} \end{array}$$

subject to

(24) $$\begin{array}{l} f_{m}\left(0\right)=0,\;\begin{array}{c} f\prime_{m}\left(0\right)=0,\;\begin{array}{c} f\prime_{m}\left(\eta \right)=0 \end{array} \end{array}\\ g_{m}\left(0\right)=0,\;\begin{array}{c} g\prime_{m}\left(0\right)=0,\;\begin{array}{c} g\prime_{m}\left(\eta \right)=0, \end{array} \end{array}\\ \theta_{m}\left(0\right)=1,\;\begin{array}{c} \theta \prime_{m}\left(\infty \right)=0 \end{array} \end{array}$$

In the formula

(25) $$\begin{array}{ll} R_{fm}\left(\eta \right)= & A_{1}\frac{\partial^{3}f_{m-1}}{\partial \eta^{3}}+\left(1+\gamma \right)\left(\sum_{n=1}^{m-1}f_{m-1-n}\frac{\partial^{2}f_{m-1}}{\partial \eta^{2}}-\sum_{n=1}^{m-1}\frac{\partial f_{m-1-n}}{\partial \eta }\frac{\partial f_{m-1}}{\partial \eta }\right)\\ & +\left(1-\gamma \right)\sum_{n=1}^{m-1}g_{m-1-n}\frac{\partial^{2}f_{m-1}}{\partial \eta^{2}}-A_{1}\sigma \frac{\partial f_{m-1}}{\partial \eta }+\left(1-C_{m}\right)\left(1+\gamma +A_{1}\sigma \right), \end{array}$$

(26) $$\begin{array}{ll} R_{gm}\left(\eta \right)= & A_{1}\frac{\partial^{3}g_{m-1}}{\partial \eta^{3}}+\left(1-\gamma \right)\left(\sum_{n=1}^{m-1}g_{m-1-n}\frac{\partial^{2}g_{m-1}}{\partial \eta^{2}}-\sum_{n=1}^{m-1}\frac{\partial g_{m-1-n}}{\partial \eta }\frac{\partial g_{m-1}}{\partial \eta }\right)\\ & +\left(1+\gamma \right)\sum_{n=1}^{m-1}f_{m-1-n}\frac{\partial^{2}g_{m-1}}{\partial \eta^{2}}-A_{1}\sigma \frac{\partial g_{m-1}}{\partial \eta }+\left(1-C_{m}\right)\left(1-\gamma +A_{1}\sigma \right), \end{array}$$

(27) $$\begin{array}{ll} R_{\theta m}\left(\eta \right)= & \frac{A_{2}}{\mathrm{Pr}}\frac{\partial^{2}\theta_{m-1}}{\partial \eta^{2}}+\left(1+\gamma \right)\left(\sum_{n=1}^{m-1}f_{m-1-n}\frac{\partial \theta_{m-1}}{\partial \eta }-\sum_{n=1}^{m-1}\theta_{m-1}\frac{\partial f_{m-1-n}}{\partial \eta }\right)\\ & +\left(1-\gamma \right)\sum_{n=1}^{m-1}\frac{\partial \theta_{m-1}}{\partial \eta }g_{m-1-n}, \end{array}$$

(28) $$C_{m}=\left\{\begin{array}{l} \begin{array}{cc} 1 & m>1 \end{array}\\ \begin{array}{cc} 0 & m=0. \end{array} \end{array}\right.$$

In Equations (25)–(27), the right-hand side is given by Equations (7)–(9), which are actually an ordinary differential equation about *η*. The particular solutions of Equations (25)–(27) are as follows:

(29) $$f_{m}^{*}\left(\eta \right)=\chi_{m}f_{m-1}\left(\eta \right)+\hbar \int_{0}^{\eta }\left\{\int_{0}^{\eta } [e^{-\beta \eta }R_{fm}\left(\eta \right)e^{\beta s}ds]d\eta \right\}d\eta .$$

(30) $$g_{m}^{*}\left(\eta \right)=\chi_{m}g_{m-1}\left(\eta \right)+\hbar \int_{0}^{\eta }\left\{\int_{0}^{\eta } [e^{-\beta \eta }R_{gm}\left(\eta \right)e^{\beta s}ds]d\eta \right\}d\eta .$$

(31) $$\theta_{m}^{*}\left(\eta \right)=\chi_{m}\theta_{m-1}\left(\eta \right)+\hbar \int_{0}^{\eta } [e^{-\beta \eta }R_{\theta m}\left(\eta \right)e^{\beta s}ds]d\eta .$$

Equation (17) leads to the following general solution:

(32) $$f_{m}\left(\eta \right)=f_{m}^{*}\left(\eta \right)+C_{0,m}+C_{1,m}\exp\left(-\beta \eta \right)+C_{2,m}\exp\left(\beta \eta \right).$$

(33) $$g_{m}\left(\eta \right)=g_{m}^{*}\left(\eta \right)+C_{3,m}+C_{4,m}\exp\left(-\beta \eta \right)+C_{5,m}\exp\left(\beta \eta \right).$$

(34) $$\theta_{m}\left(\eta \right)=\theta_{m}^{*}\left(\eta \right)+C_{6,m}\exp\left(-\beta \eta \right)+C_{7,m}\exp\left(\beta \eta \right).$$

Consequently, an infinite number of linear ordinary differential Equation (23) have constant coefficients derived from the original nonlinear partial differential Equations (7)–(9). The solution of nonlinear partial differential equations involving variable coefficients is undoubtedly easier than solving linear ordinary differential equations involving constant coefficients. There is no requirement for any small or large physical parameters in this transformation, unlike the perturbation method. There is no obvious relationship between the linear terms in the original Equations (7)–(9) and the auxiliary linear Operator (16). The main reason for this is that the homotopy analytic method is very flexible in terms of auxiliary linear operators, unlike other analytical methods. The linear ordinary differential Equation (23) with variable coefficients cannot be converted into the nonlinear coupled ordinary differential Equations (7)–(9) without this degree of freedom. The following series solutions of Equations (7)–(9) can be obtained.

(35) $$f\left(\eta \right)=f_{0}\left(\eta \right)+\sum_{m=1}^{+\infty }f_{m}\left(\eta \right),g\left(\eta \right)=g_{0}\left(\eta \right)+\sum_{m=1}^{+\infty }g_{m}\left(\eta \right),\theta \left(\eta \right)=\theta_{0}\left(\eta \right)+\sum_{m=1}^{+\infty }\theta_{m}\left(\eta \right).$$

noting that $f\left(\eta \right),g\left(\eta \right),\theta \left(\eta \right)$ given by the homotopy analysis method contain three unknown convergence control parameters $h_{f},h_{g},h_{\theta }$. The series solution relies on these to ensure convergence. The mean residual errors of the kth order approximations are defined as follows:

(36) $$\varepsilon_{k}^{f}\left(h_{f},h_{g},h_{\theta }\right)=\frac{1}{N+1}{\sum_{i=0}^{N} [{\left.N_{f}\left(\sum_{m=0}^{k}f_{m}\right)\right|}_{\eta =i\beta \eta }]}^{2},$$

(37) $$\varepsilon_{k}^{g}\left(h_{f},h_{g},h_{\theta }\right)=\frac{1}{N+1}{\sum_{i=0}^{N} [{\left.N_{g}\left(\sum_{m=0}^{k}f_{m},\sum_{m=0}^{k}g_{m}\right)\right|}_{\eta =i\beta \eta }]}^{2},$$

(38) $$\varepsilon_{k}^{\theta }\left(h_{f},h_{g},h_{\theta }\right)=\frac{1}{N+1}{\sum_{i=0}^{N} [{\left.N_{\theta }\left(\sum_{m=0}^{k}f_{m},\sum_{m=0}^{k}g_{m},\sum_{m=0}^{k}\theta_{m}\right)\right|}_{\eta =i\beta \eta }]}^{2},$$

For the original governing Equations (7)–(9), an approximation of the *k*th order has a total error defined as follows:

(39) $$\varepsilon_{k}^{tol}\left(h_{f},h_{g},h_{\theta }\right)=\varepsilon_{k}^{f}\left(h_{f},h_{g},h_{\theta }\right)+\varepsilon_{k}^{g}\left(h_{f},h_{g},h_{\theta }\right)+\varepsilon_{k}^{\theta }\left(h_{f},h_{g},h_{\theta }\right).$$

As a result of the kth order approximation, the optimal value for $h_{f},h_{g},h_{\theta }$ can be determined as the minimum of total error of $\varepsilon_{k}^{tol}$.

## 3. Results Analysis and Discussion

**Figure 2:**
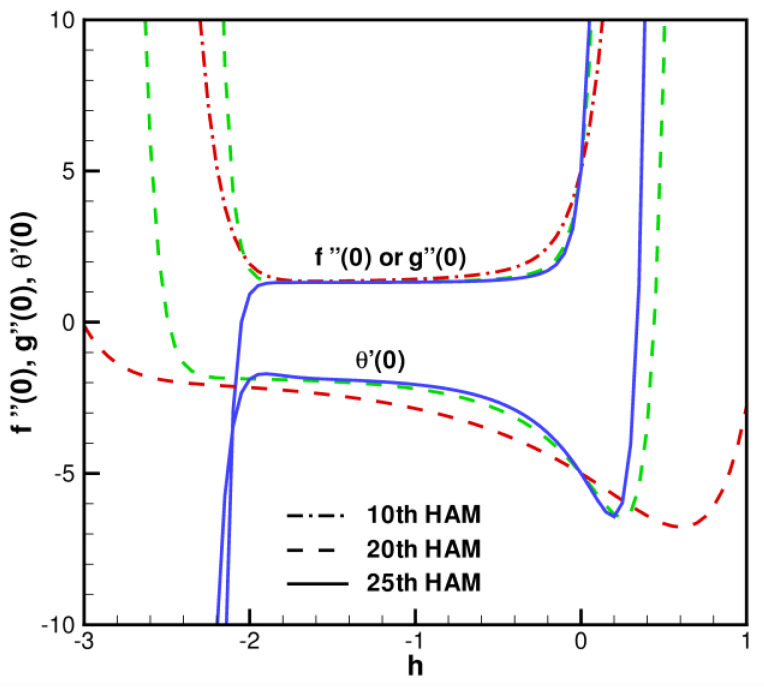
Curves $f_{\eta \eta }\left(\eta \right),g_{\eta }\left(\eta \right)$ change with *h* when β=5, η=0.

Based on the HAM results, Cu-Al_2_O_3_-H_2_O spherical hybrid nanoparticles for Homann stagnation-point flows of flat sheets in porous media are solved approximately and analytically. The convergence control parameter *h* and auxiliary parameter *β* in the series Solution (35) ensure the convergence of order solutions. By examining the curves of the control parameters, we are able to determine the optimal range for the auxiliary control parameter, which is crucial to ensuring that the series solution converges. Therefore, if the series Solution (35) is converging, it follows that the derivative $f_{\eta \eta }\left(\eta \right),g_{\eta \eta }\left(\eta \right),\theta_{\eta }\left(\eta \right)$ must also be converging. In order to simplify things, consider the convergence of the $f_{\eta \eta }\left(\eta \right),g_{\eta \eta }\left(\eta \right),\theta_{\eta }\left(\eta \right)$ series solutions. Because the original nonlinear partial differential Equations (7)–(9) are coupled, *β* must be greater than 2. Unless *β* and *η* are fixed, series solutions of $f_{\eta \eta }\left(\eta \right),g_{\eta \eta }\left(\eta \right),\theta_{\eta }\left(\eta \right)$ are only dependent on control parameter *h*. As shown in Figure 2, let β=5, and $f_{\eta \eta }\left(\eta \right),g_{\eta \eta }\left(\eta \right),\theta_{\eta }\left(\eta \right)$ are just power series of *h* whose convergence is dependent upon *h* when *η* = 0. There are two points that need to be emphasized. A first benefit of the HAM method is that it is a superior method to other analytical and semi-analytic methods. Nonlinear problems with strong nonlinearity can be converged accurately with HAM. In spite of large perturbations, HAM maintains independence from small or large physical parameters and presents convenient control over homotopy series solutions. A second advantage is that it converges rapidly when the convergence control parameters are optimized. With an increasing order of approximation, the residual total decreases for each case.

**Figure 3:**
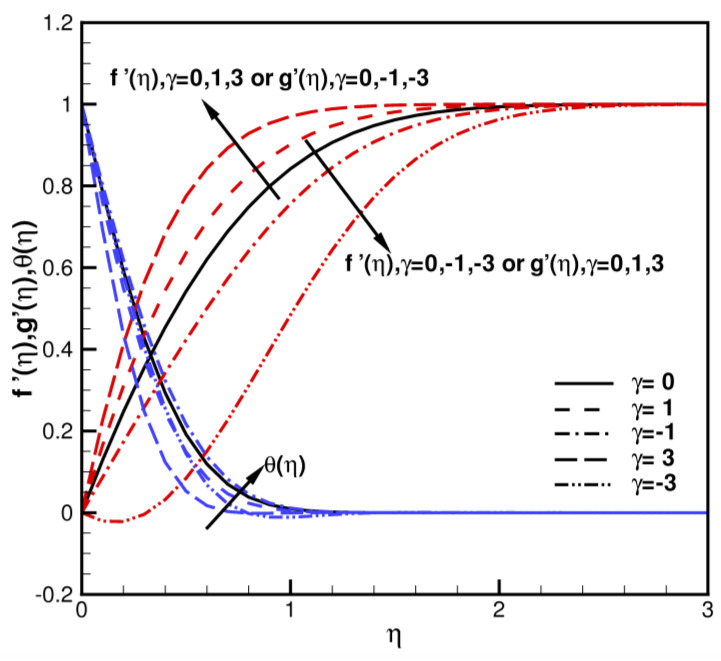
The dimensionless velocities and temperature distributions of $f^{\prime }\left(\eta \right),g^{\prime }\left(\eta \right),\theta \left(\eta \right)$ when $\phi =\phi_{1}=\phi_{2}$ = 0, *σ* = 0, and Pr = 6.2.

**Figure 4:**
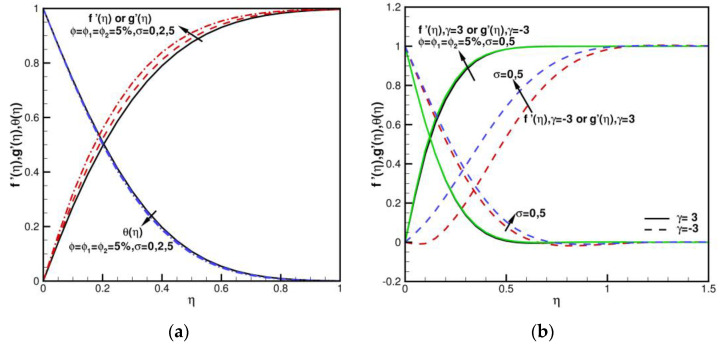
The dimensionless velocities and temperature distributions of $f^{\prime }\left(\eta \right),g^{\prime }\left(\eta \right),\theta \left(\eta \right)$ when $\phi =\phi_{1}=\phi_{2}$ = 5% and Pr = 6.2. (**a**) Impact of *γ* = 0, *σ* = 0, 2, 5; (**b**) Impact of *γ* = −3, 3, *σ* = 0, 5.

The analysis and discussion of results obtained using HAM is as follows. The analysis involves a discussion of the impact of many physical parameters generated in the proposed model. When *γ* = 0, $\phi =\phi_{1}=\phi_{2}=0$, and *σ* = 0, it can be found that $f\left(\eta \right)=g\left(\eta \right)$ represents the axisymmetric Homann stagnation-point flow. When *γ* = 0 (axisymmetric), $\phi =\phi_{1}=\phi_{2}=0$ (regular fluid), and σ=0 (non-porous medium), $f^{\prime \prime }\left(0\right)$ = 1.311608, compared with the $f^{\prime \prime }\left(0\right)$ = 1.311938 of Waini et al. [40], Wang [63], and Soid et al. [64], and the relative error is not more than 0.025%. Table 3 presents a comparison of corresponding values of $f_{\eta \eta }\left(0\right)$ and $\theta_{\eta }\left(0\right)$ with *γ*, ϕ, *σ*. A 20th order homotopy solution is adopted without higher order calculation, and the convergence result is accurate enough, especially for the large values of *γ*, ϕ, and *σ*. As shown in Table 3, skin friction coefficients $Cf_{x},Cf_{y}$ and Nusselt number $Nu_{x}$ with various values of *γ*, ϕ, *σ* when Pr = 6.2 are calculated and compared with the results of Waini et al. [40]. The consistency of results is very good. When Pr = 6.2, *σ* = 0, and the volume fraction $\phi =\phi_{1}=\phi_{2}=0$, the velocities of $f^{\prime }\left(\eta \right),g^{\prime }\left(\eta \right)$ and the temperature $\theta \left(\eta \right)$ distributions under different values of shear-strain rate ratios *γ* = 0, ±1, ±3 are presented in Figure 2. The behaviors of flow fields by changing the shear–strain rate ratios *γ* are studied. When γ>0, $f^{\prime }\left(\eta \right),g^{\prime }\left(\eta \right)$ increase with an increase in *γ*; $\theta \left(\eta \right)$ decreases with an increase in *γ*. When γ<0, $f^{\prime }\left(\eta \right),g^{\prime }\left(\eta \right)$ decrease as *γ* decreases; $\theta \left(\eta \right)$ decreases with the decrease in *γ* generally. $f^{\prime }\left(\eta \right)$ flows in the reverse direction towards the plate at γ=−3, or $g^{\prime }\left(\eta \right)$ flows in the reverse direction towards the plate at *γ* = 3. Flows are inward near the stagnation zone, as shown in Figure 3. When Pr = 6.2 and $\phi =\phi_{1}=\phi_{2}=5\%$, the velocities of $f^{\prime }\left(\eta \right),g^{\prime }\left(\eta \right)$ and the temperature $\theta \left(\eta \right)$ curves with different *γ* and *σ* values are shown in Figure 4a,b. $f^{\prime }\left(\eta \right),g^{\prime }\left(\eta \right)$, $\theta \left(\eta \right)$ increase as *σ* increases. When the volume fraction $\phi ,\phi_{1},\phi_{2}$ of spherical hybrid nanoparticles Cu-Al_2_O_3_-H_2_O increases, the coefficients of skin friction and local Nusselt numbers increase. The Prandtl number is fixed at 6.2, and does not consider the variations.

**Figure 5:**
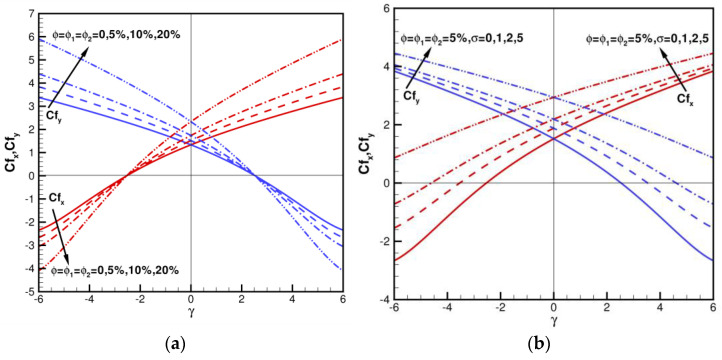
The skin friction coefficients of $Cf_{x},Cf_{y}$ for various *γ*, ϕ, and *σ* when Pr = 6.2. (**a**) Impact of *σ* = 0, $\phi =\phi_{1}=\phi_{2}$ = 0, 5%, 10%, 20%; (**b**) Impact of $\phi =\phi_{1}=\phi_{2}$ = 5%, *σ* = 0, 1, 2, 5.

Figure 5a,b illustrates variations in the skin friction coefficients $Cf_{x},Cf_{y}$ with *γ* in the range of −6≤γ≤6 for various $\phi =\phi_{1}=\phi_{2}=0$, 5%, 10%, 20%, and *σ* = 0, 1, 2, 5 when Pr = 6.2. $Cf_{x},Cf_{y}$ show a symmetric pattern, where the line of symmetry lies at *γ* = 0 in the axisymmetric case. When *γ* = 0, *σ* = 0, and $\phi =\phi_{1}=\phi_{2}=0$, $Cf_{x}=f^{\prime \prime }\left(0\right)$ = 1.311608 and $Cf_{y}=g^{\prime \prime }\left(0\right)$ = 1.311608, also as shown in Table 3. As shown in Figure 2a, $Cf_{x}=Cf_{y}$ = 0, and the shear stress value is 0 when γ=±2.50319. When γ<−2.503193, $Cf_{x}$ decreases with $\phi ,\phi_{1},\phi_{2}$ increasing; when γ>−2.50319, $Cf_{x}$ increases with $\phi ,\phi_{1},\phi_{2}$ increasing. When γ>2.50319, $Cf_{y}$ decreases with $\phi ,\phi_{1},\phi_{2}$ increasing; when γ<2.50319, $Cf_{y}$ increases with $\phi ,\phi_{1},\phi_{2}$ increasing. Furthermore, for the axisymmetric case of *γ* = 0, when the volume fraction $\phi =\phi_{1}=\phi_{2}=0$, 5%, 10%, 20%, $Cf_{x}=Cf_{y}$ = 1.33634, 1.51918, 1.73905, 2.33449. Compared with $\phi =\phi_{1}=\phi_{2}=0$, it can be found that the wall shear stress values increase by 13.68%, 30.14%, and 74.69%, respectively. It is shown that the percentage of skin friction coefficient is enhanced by hybrid nanofluid relative to regular fluid. By increasing the fraction of hybrid nanofluids in the fluid, the wall shear stress can be increased. Trends of $Cf_{x}$ are opposite for $Cf_{y}$. When $\phi =\phi_{1}=\phi_{2}$ = 5%, Pr = 6.2, variations of skin friction coefficients $Cf_{x},Cf_{y}$ in range of −6≤γ≤6 for various *σ* = 0, 1, 2, 5 are shown in Figure 2b. Moreover, for the axisymmetric case of *γ* = 0, when volume fraction $\phi =\phi_{1}=\phi_{2}$ = 5%, $Cf_{x}=Cf_{y}$ = 1.51918, 1.87869, 2.18885, 2.93963 for various *σ* = 0, 1, 2, 5. Compared with $\phi =\phi_{1}=\phi_{2}$ = 5%, *σ* = 0, it can be found that shear stress values increase 23.66%, 40.08%, 93.50%, respectively. It shows that shear stress can be enhanced by increasing the coefficient of permeability in porous media. As *σ* increases, $Cf_{x}$ and $Cf_{y}$ increase. When ϕ, *γ* are given certain values, the greater the values of *σ*, the stronger $Cf_{x}$ and $Cf_{y}$.

**Figure 6:**
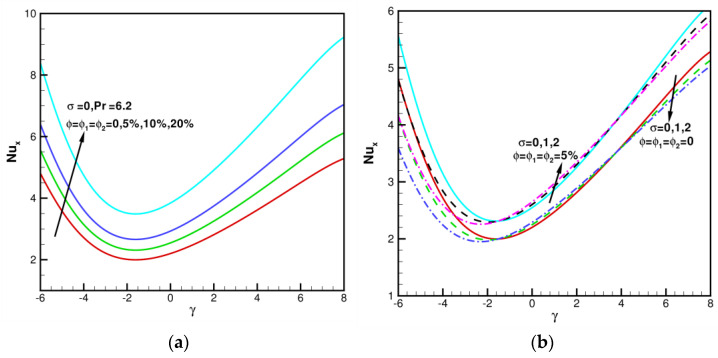
Local Nusselt number $Nu_{x}$ for various γ, ϕ, and *σ* when Pr = 6.2. (**a**) Impact of *σ* = 0, $\phi =\phi_{1}=\phi_{2}$ = 0, 5%, 10%, 20%; (**b**) Impact of $\phi =\phi_{1}=\phi_{2}$ = 0, 5%, *σ* = 0, 1, 2.

Figure 6a,b illustrates that variations in local Nusselt number $Nu_{x}$ against *γ* in the range of −6≤γ≤8 for various $\phi =\phi_{1}=\phi_{2}=0$, 5%, 10%, 20%, and *σ* = 0, 1, 2 when Pr = 6.2. As shown in Figure 3a, when $\phi =\phi_{1}=\phi_{2}=0$, 5%, 10%, 20%, *σ* = 0, and Pr = 6.2, $Nu_{x}$ increases as the value of $\phi ,\phi_{1},\phi_{2}$ increases. When *γ* < 0, the values of $Nu_{x}$ increase steeply with volume fractions $\phi ,\phi_{1},\phi_{2}$ increasing. When *γ* > 0, the values of $Nu_{x}$ increase and slow down with volume fractions $\phi ,\phi_{1},\phi_{2}$ increasing. When $\phi =\phi_{1}=\phi_{2}$ = 0, 5%, and Pr = 6.2, $Nu_{x}$ against *γ* in the range of −6≤γ≤8 for various *σ* = 0, 1, 2 are shown in Figure 5b. As $\phi ,\phi_{1},\phi_{2}$ increase, the values of $Nu_{x}$ increase. When *γ* < 0, values of $Nu_{x}$ decrease and change clearly with *σ* increasing. When *γ* > 0, values of $Nu_{x}$ are less affected by the values of *σ*.

## 4. Conclusions

Non-axisymmetric Homann stagnant-point flows of the flat plate in porous media containing spherical Cu-Al_2_O_3_-H_2_O nanoparticles are studied using the homotopy analysis method (HAM). The procedure for solving nonlinear coupled ordinary differential equations with variable coefficients involves transforming them into linear ordinary differential equations with constant coefficients. Analytical solutions can be obtained for these equations across the whole domain. A similarity transformation is applied to the governing equations in order to convert them into three coupled nonlinear ordinary differential equations. The analysis involves a discussion of impact of many physical parameters generated in the proposed model. The results have shown that the skin friction coefficients of *Cf_x_*_,_ *Cf_y_* increase with the volume fraction of the hybrid nanofluid and the coefficient of permeability in porous media. For the axisymmetric case of *γ* = 0, when volume fraction φ = φ_1_ = φ_2_ = 0, 5%, 10%, 20%, *Cf_x_* = *Cf_y_* = 1.33634, 1.51918, 1.73905, 2.33449. Compared with φ = φ_1_ = φ_2_ = 0, it can be found that the wall shear stress values increase by 13.68%, 30.14%, 74.69%, respectively. Local Nusselt numbers *Nu_x_* increase as the hybrid nanofluid volume fraction increases. The values of *Nu_x_* decrease, and change clearly with the coefficient of permeability increasing in the range of *γ* < 0; otherwise, Nu_x_ are less affected in the range of *γ* > 0. The shape of nanoparticles has an important effect on the thermal and flow characteristics of nanofluids or hybrid nanofluids. Future works will involve the shape factor of nanoparticles and the mass-based hybrid nanofluid model in addition to the present research.

## Figures and Tables

**Table 1:** Nanoparticles and H_2_O thermophysical properties [40].

Type	$\rho \;({\mathrm{kg}/m}^{3})$	*k* (W/mK)	$c_{p}\;(J/\mathrm{kgK})$
H_2_O	997.1	0.613	4179
Cu	8933	401	385
Al_2_O_3_	3970	40	765

**Table 2:** Applied model for thermophysical properties of spherical hybrid nanoparticles [40].

Property	Mathematical Relations
Density	$\rho_{hnf}=\phi_{1}\rho_{n1}+\phi_{2}\rho_{n2}+\left(1-\phi \right)\rho_{f}$
Heat capacity	${\left(\rho c_{p}\right)}_{hnf}=\phi_{1}{\left(\rho c_{p}\right)}_{n1}+\phi_{2}{\left(\rho c_{p}\right)}_{n2}+\left(1-\phi \right){\left(\rho c_{p}\right)}_{f}$
Dynamic viscosity	$\mu_{hnf}=\frac{\mu }{{\left(1-\phi \right)}^{2.5}}$
Thermal conductivity	$\frac{k_{hnf}}{k_{f}}=\frac{\frac{\phi_{1}k_{n1}+\phi_{2}k_{n2}}{\phi }+2k_{f}+2\left(\phi_{1}k_{n1}+\phi_{2}k_{n2}\right)-2\phi k_{f}}{\frac{\phi_{1}k_{n1}+\phi_{2}k_{n2}}{\phi }+2k_{f}-\left(\phi_{1}k_{n1}+\phi_{2}k_{n2}\right)+\phi k_{f}}$

**Table 3:** Skin friction coefficients $Cf_{x},Cf_{y}$ and Nusselt numbers $Nu_{x}$ with various values of *γ*, ϕ and *σ* when Pr = 6.2.

γ	φ	σ	$Cf_{x}$ (Refs. [40,62,63])	HAM 20th	$Cf_{y}$ (Refs. [40,62,63])	HAM 20th	$Nu_{x}$ (Refs. [40,62,63])	HAM 20th
0	0	0	1.311938	1.311608	1.311938	1.311608	1.806069	1.810147
5			3.038940	3.036096	−0.894909	−0.902242	3.938146	3.998352
−5			−0.894909	−0.902242	3.038940	3.036096	3.074275	3.084240
−5	2%		−0.966699	−0.966456	3.282727	3.281901	3.203682	3.210915
	4%		−1.039700	−1.039438	3.530622	3.529834	3.330939	3.669205
	2%	2	−0.027231	−0.027224	3.569397	3.568499	2.788727	2.795023

## Data Availability

The manuscript includes all relevant data.

## Nomenclature

$\bar{u},\bar{v},\bar{w}$	the velocity components
${\bar{u}}_{e},{\bar{v}}_{e},{\bar{w}}_{e}$	the external flow velocities
$\bar{a},\bar{b}$	the strain shear rates
${\bar{T}}_{\infty }$	the ambient temperature
${\bar{T}}_{0}$	the characteristic temperature
$\bar{K}$	the permeability of porous media
$\bar{k}$	the conductivity in temperature
$c_{p}$	the coefficient of specific heat capacity
$Cf_{x},Cf_{y}$	the coefficients of skin friction
$Nu_{x}$	the Nusselt number
$A_{1},A_{2}$	the coefficients from hybrid nanofluids
Pr	the Prandtl number
Rex,Rey	the local Reynolds numbers
**Greek symbols**	
ρ	the fluid density
*μ*	the dynamic viscosity
$\phi ,\phi_{1},\phi_{2}$	the volume fraction of hybrid nanofluids
*γ*	the ratio of shear–strain rate
*σ*	the coefficient of permeability
